# Vibrio cholerae Sialidase-Specific Immune Responses Are Associated with Protection against Cholera

**DOI:** 10.1128/mSphere.01232-20

**Published:** 2021-04-28

**Authors:** M. Hasanul Kaisar, Mohammed Saruar Bhuiyan, Aklima Akter, Danial Saleem, Anita S. Iyer, Pinki Dash, Al Hakim, Fahima Chowdhury, Ashraful Islam Khan, Stephen B. Calderwood, Jason B. Harris, Edward T. Ryan, Firdausi Qadri, Richelle C. Charles, Taufiqur Rahman Bhuiyan

**Affiliations:** a Infectious Diseases Division, icddr,b (International Centre for Diarrhoeal Disease Research, Bangladesh), Dhaka, Bangladesh; b College of Physicians and Surgeons, Columbia University, New York, New York, USA; c Division of Infectious Diseases, Massachusetts General Hospital, Boston, Massachusetts, USA; d Department of Medicine, Harvard Medical School, Boston, Massachusetts, USA; e Department of Microbiology, Harvard Medical School, Boston, Massachusetts, USA; f Division of Global Health, Massachusetts General Hospital for Children, Boston, Massachusetts, USA; g Department of Pediatrics, Harvard Medical School, Boston, Massachusetts, USA; h Department of Immunology and Infectious Diseases, Harvard School of Public Health, Boston, Massachusetts, USA; UTMB

**Keywords:** sialidase, cholera, immune response, *Vibrio cholerae*, antibody, humoral, mucosal, children, adults, memory B cell

## Abstract

Cholera remains a major public health problem in resource-limited countries. Vaccination is an important strategy to prevent cholera, but currently available vaccines provide only 3 to 5 years of protection. Understanding immune responses to cholera antigens in naturally infected individuals may elucidate which of these are key to longer-term protection seen following infection. We recently identified Vibrio cholerae O1 sialidase, a neuraminidase that facilitates binding of cholera toxin to intestinal epithelial cells, as immunogenic following infection in two recent high-throughput screens. Here, we present systemic, mucosal, and memory immune responses to sialidase in cholera index cases and evaluated whether systemic responses to sialidase correlated with protection using a cohort of household contacts. Overall, we found age-related differences in antisialidase immune response following cholera. Adults developed significant plasma anti-sialidase IgA, IgG, and IgM responses following infection, whereas older children (≥5 years) developed both IgG and IgM responses, and younger children only developed IgM responses. Neither older children nor younger children had a rise in IgA responses over the convalescent phase of infection (day 7/day 30). On evaluation of mucosal responses and memory B-cell responses to sialidase, we found adults developed IgA antibody-secreting cell (ASC) and memory B-cell responses. Finally, in household contacts, the presence of serum anti-sialidase IgA, IgG, and IgM antibodies at enrollment was associated with a decrease in the risk of subsequent infection. These data show cholera patients develop age-related immune responses against sialidase and suggest that immune responses that target sialidase may contribute to protective immunity against cholera.

**IMPORTANCE** Cholera infection can result in severe dehydration that may lead to death within a short period of time if not treated immediately. Vaccination is an important strategy to prevent the disease. Oral cholera vaccines provide 3 to 5 years of protection, with 60% protective efficacy, while natural infection provides longer-term protection than vaccination. Understanding the immune responses after natural infection is important to better understand immune responses to antigens that mediate longer-term protection. Sialidase is a neuraminidase that facilitates binding of cholera toxin to intestinal epithelial cells. We show here that patients with cholera develop systemic, mucosal, and memory B-cell immune responses to the sialidase antigen of Vibrio cholerae O1 and that plasma responses targeting this antigen correlate with protection.

## Abstract (precis)

Cholera infection can result in severe dehydration that may lead to death within a short period of time if not treated immediately. Vaccination is an important strategy to prevent the disease.

## INTRODUCTION

Cholera, a severe dehydrating diarrheal disease, remains an important public health problem in resource-limited countries, particularly in certain parts of Asia and Africa, where significant sections of the population lack access to safe water and adequate sanitation. In addition, natural disasters, such as the earthquake in Haiti in 2010, and conflicts, such as the ongoing war in Yemen, have been accompanied by epidemics of cholera (1–3). The WHO estimates that there are approximately 3 million cases of cholera worldwide each year, resulting in about 95,000 deaths (4). An important strategy to prevent and control cholera is vaccination. However, current vaccines provide more limited protection, especially in younger age groups (5–7), compared to natural infection with Vibrio cholerae, which induces 90 to 100% protection against reinfection that lasts for up to 10 years in adults and children (8, 9). Understanding how long-lasting protection after natural infection is mediated may provide insights on how to improve current vaccine efficacy and duration of protection.

Recently, we have shown in two separate immunoscreens using antigen arrays containing nearly all the V. cholerae O1 proteins that adult cholera patients develop antibody responses against the V. cholerae sialidase during the early convalescent stages (10, 11). The first study characterized the antigenic targets of a library of monoclonal antibodies generated by single-cell expression of cholera-induced antibody-secreting cells and identified the V. cholerae sialidase as the third most common target after V. cholerae O-specific polysaccharide (OSP) and cholera toxin (CT) (10). This has been further supported by a second study, which found high immunoreactivity in plasma and antibody-in-lymphocyte supernatant (ALS) from adult cholera patients to the V. cholerae sialidase (11).

V. cholerae sialidase, also known as neuraminidase, is a virulence factor that catalyzes the cleavage of terminal sialic acid residues from glycoproteins and glycolipids. V. cholerae sialidase can hydrolyze both α-2,3- and α-2,6-linked glycosidic bonds and has an essential Ca^2+^ ion, making it unique from other microbial sialidases (12–14). It is encoded by the *nanH* gene, which is found in the 57-kb pathogenicity island (VPI-2) and is located directly downstream of the genes involved in the transport and catabolism of sialic acid (15, 16). Sialdiase may play an important role in the pathogenesis of V. cholerae for two reasons. First, it removes sialic acid residues from higher-order gangliosides on the membranes of gut epithelial cells to generate monogangliosides (GM1), the binding site for CT (17). We have previously demonstrated that sialidase treatment potentiated the effect of CT-induced cAMP production on cultured human colorectal Caco-2 cell lines, and antibodies targeting sialidase neutralized this effect in a dose-dependent manner (10). Second, sialidase may help V. cholerae colonize heavily sialylated areas like the intestinal epithelium; the free sialic acid residues released by sialidase may serve as carbon and energy sources for V. cholerae. A study employing infant mouse models demonstrated that wild-type V. cholerae strains experienced a significant growth advantage in the early stages of infection compared to knockout strains that could not utilize sialic acid (18).

Taken together, these observations raise the possibility that immune responses to sialidase might potentially play a role in protection against cholera. Hence, we have investigated here more systematically the serologic, mucosal, and memory B-cell responses to sialidase in patients hospitalized with cholera in Dhaka, Bangladesh, and utilized a cohort of household contacts of cholera index cases to evaluate whether plasma responses to sialidase on exposure correlate with protection against cholera.

## RESULTS

### Study population.

We enrolled 18 younger children (≤5 years of age, median age 4 years), 29 older children (6 to 17 years of age, median age 10 years), and 31 adults (18 to 55 years of age, median age 28 years), each admitted with dehydrating diarrhea to the icddr,b hospital, Dhaka, with culture-confirmed cholera without any other copathogens present (19, 20). All patients were infected with the V. cholerae O1 El Tor biotype of either the Ogawa or Inaba serotype. The baseline demographic and clinical characteristics of the cholera cases are shown in Table 1.

**TABLE 1:** Characteristics of cholera patients enrolled in the study

Characteristics	Result for:					
	Ogawa (*n* = 37)			Inaba (*n* = 41)		
	Younger children (2–5 yr, *n* = 14)	Older children (6–17 yr, *n* = 16)	Adults (18–55 yr, *n* = 7)	Younger children (2–5 yr, *n* = 4)	Older children (6–17 yr, *n* = 13)	Adults (18–55 yr, *n* = 24)
Female, no. (%)	4 (28.6)	6 (37.5)	3 (42.9)	1 (25)	3 (23.1)	11 (45.8)
Median age, yr (25th, 75th percentile)	4 (3, 4.8)	11 (9.3, 13)	26 (24, 30)	4 (3.75, 4)	9 (8, 10)	29 (22.8, 33)
Blood group O, no. (%)	5 (35.7)	9 (56.3)	5 (71.4)	2 (50)	4 (30.8)	10 (41.7)

For the household contacts, we included 418 household contacts of 98 index patients with culture-confirmed V. cholerae O1 serotype Ogawa enrolled from 2001 to 2015: 131 were categorized as subsequently infected, and 287 were categorized as uninfected. The baseline demographic and clinical characteristics of the household contacts are shown in Table 2.

**TABLE 2:** Characteristics of household contacts enrolled in the study

Characteristics	Result for:	
	Infected (*n* = 131)	Uninfected (*n* = 283)
Female, no. (%)	71 (54.2)	150 (53)
Median age, yr (25th, 75th percentile)	24.8 (9.3, 35.0)	27.4 (14.4, 36.3)
Blood group O, no. (%)	41 (31.3)	101 (35.7)

### Vibriocidal antibody responses.

**FIG 1:**
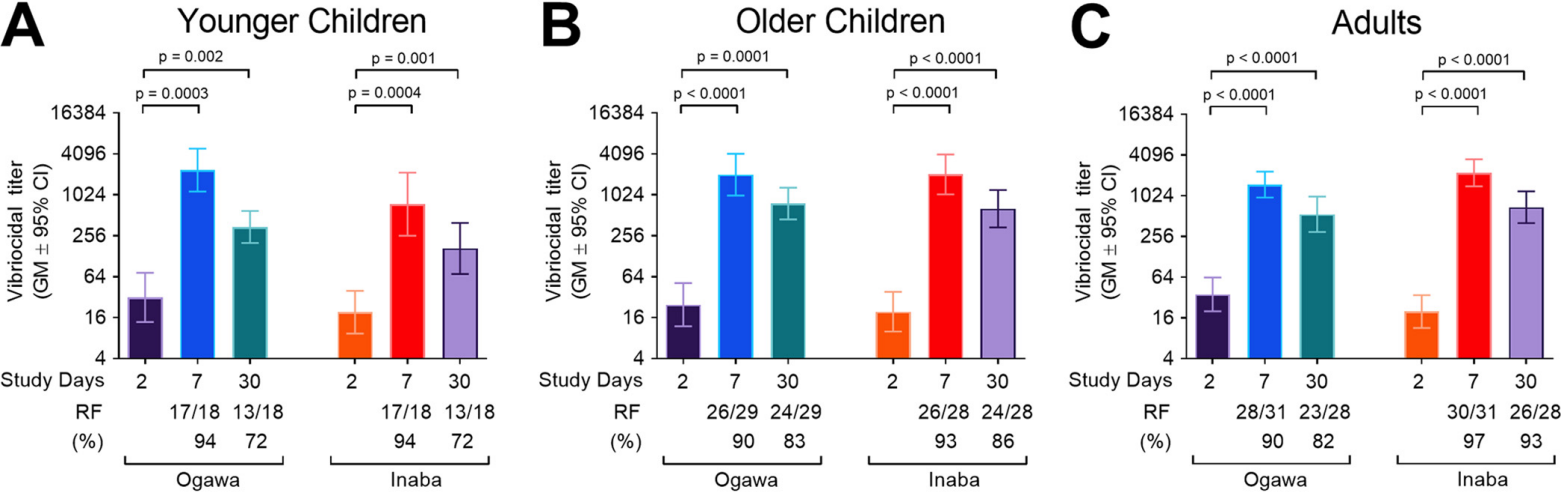
Vibriocidal antibody responses and responder frequencies by age. The geometric mean vibriocidal titers to V. cholerae O1 Ogawa (strain X25049) and Inaba (strain T19479) are displayed for days 2, 7, and 30 in younger children (A), older children (B), and adults (C) with cholera. Differences within groups were assessed using the Wilcoxon matched-pairs signed-rank test. *P ≤ *0.05 was considered significant. Responder frequency (RF; percentage of patients with a ≥4-fold rise in titer) is displayed below the *x* axis.

We measured vibriocidal antibody titers on days 2 (acute), 7 (early convalescent) and 30 (late convalescent) in the patients with cholera (Fig. 1). Vibriocidal antibody responses peaked on day 7 compared to baseline and remained elevated on day 30 in all 3 age cohorts (Fig. 1). Only one patient failed to have a 4-fold or greater increase in vibriocidal antibody titers (with either Ogawa or Inaba) from the acute to the convalescent phases of illness, likely secondary to elevated baseline titer (640 for Ogawa; 80 for Inaba).

### Antisialidase antibody responses in plasma.

**FIG 2:**
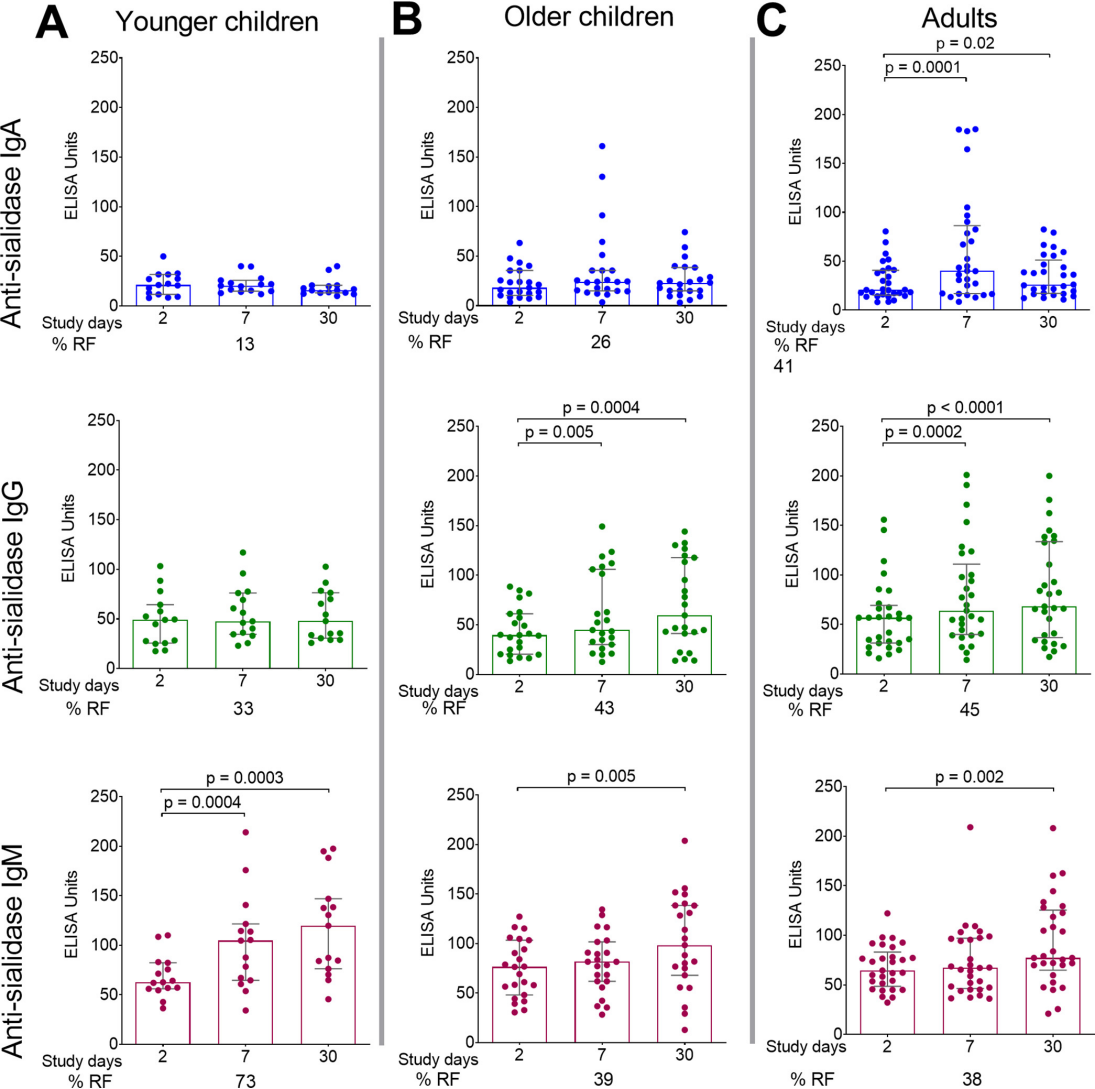
Plasma sialidase-specific responses by age. Shown are anti-sialidase IgA, IgG, and IgM plasma responses in younger children (A), older children (B), and adults (C) with cholera on days 2, 7, and 30. Medians with interquartile ranges are shown. Differences within groups were assessed using the Wilcoxon matched-pairs signed rank test. *P ≤ *0.05 was considered significant. RF, responder frequency (percentage of patients with a 1.5-fold increase from baseline to convalescence [day 7 and/or day 30]).

We compared sialidase-specific antibody responses in plasma at the acute phase of infection (day 2) to the convalescent phase of infection (days 7 and 30) in all 3 age cohorts (Fig. 2). Adults had increased responses on days 7 and 30 for IgA (*P* = 0.0001 and *P* = 0.02) and IgG (*P* = 0.0002 and *P* < 0.0001), but an increase in anti-sialidase IgM responses was not observed until day 30 (*P* = 0.002). Older children had increased IgG responses on days 7 and 30 (*P* = 0.005 on *P* = 0.0004) and IgM by day 30 (*P* = 0.005); however, they did not have significant IgA responses compared to day 2. In younger children (under 5 years of age), there were increased IgM responses on both days 7 (*P* = 0.0004) and 30 (*P* = 0.0003) compared to day 2, but no significant increase in IgA or IgG responses were observed on day 7 or 30. This is in contrast to antibody responses to cholera toxin B (CTB), where all age groups had significant IgA and IgG responses by days 7 and 30, and no age groups had a significant IgM response (see Fig. S1 in the supplemental material).

10.1128/mSphere.01232-20.1 FIG S1 Plasma CTB-specific responses by age. Anti-CTB IgA, IgG, and IgM plasma responses in younger children (A), older children (B), and adults (C) with cholera on days 2, 7, and 30. Medians with interquartile ranges are shown. Differences within groups were assessed using the Wilcoxon matched-pairs signed-rank test. *P ≤ *0.05 was considered significant. RF, responder frequency (percentage of patients with 1.5-fold increase from baseline to convalescence on day 7 and/or day 30). Download FIG S1, TIF file, 0.1 MB. Copyright © 2021 Kaisar et al. 2021 Kaisar et al. https://creativecommons.org/licenses/by/4.0/ This content is distributed under the terms of the Creative Commons Attribution 4.0 International license.

### Antibody-secreting cell responses as a marker of mucosal immune responses.

**FIG 3:**
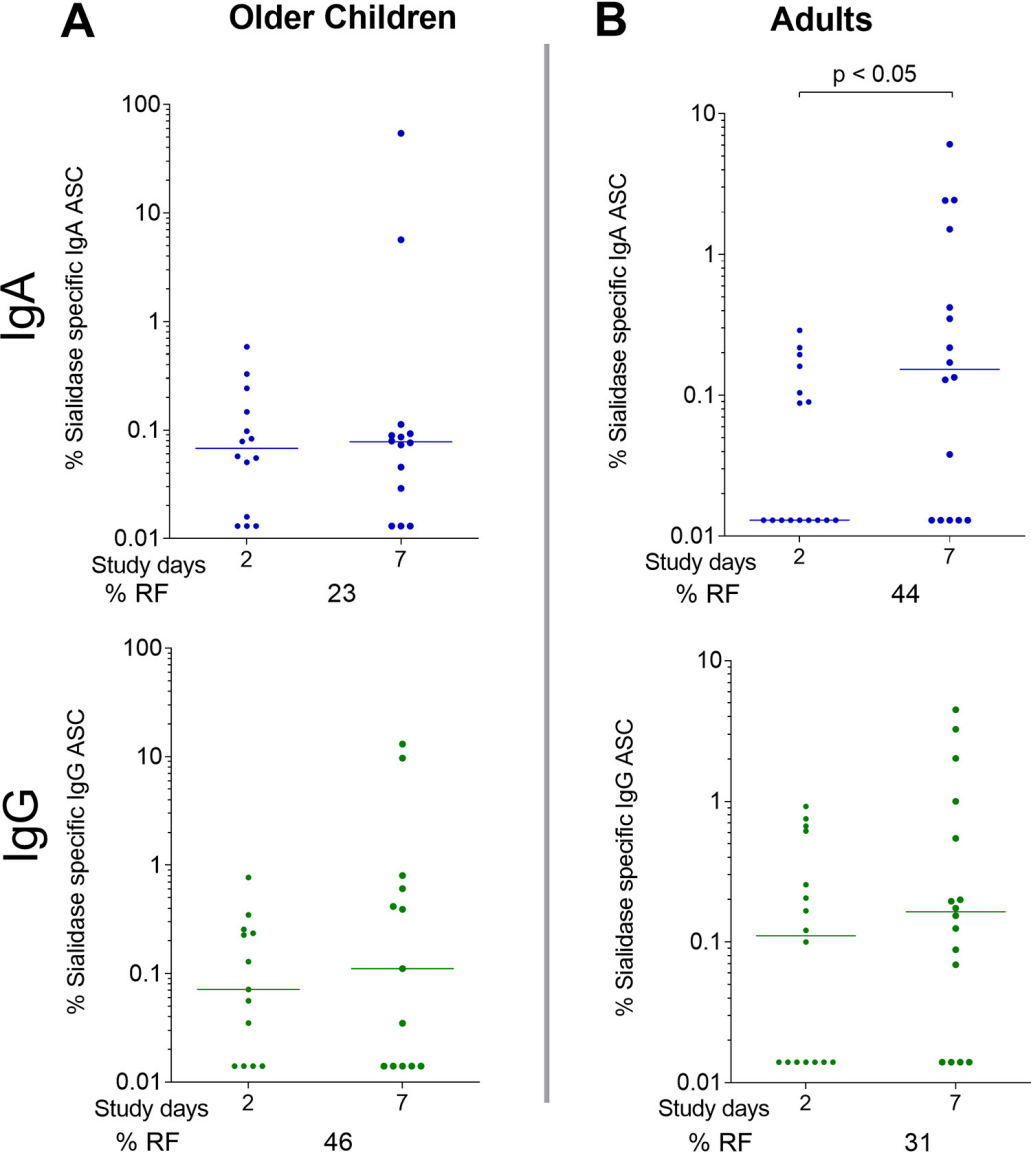
Sialidase-specific IgA and IgG antibody-secreting cell (ASC) responses in patients with cholera. Shown are the median proportions of anti-sialidase IgA and IgG ASCs as a fraction of the total ASCs of same antibody isotype in older children (A) and adults (B) on days 2 and 7 following cholera. Differences within groups were assessed using the Wilcoxon matched-pairs signed-rank test. *P ≤ *0.05 was considered significant. RF, responder frequency (percentage of patients with a 4-fold increase at convalescence [day 7 and/or day 30]).

We measured mucosa-derived sialidase-specific IgA- and IgG-producing antibody-secreting cell (ASC) responses on days 2 and 7 in adults (*n* = 16) and older children (*n* = 13); younger children were excluded due to the limited blood volume available for isolation of peripheral blood mononuclear cells (PBMCs) (Fig. 3). In adults, there were significant IgA (*P* < 0.05) ASC responses on day 7 compared to day 2. In older children, there was not a significant increase in IgA or IgG ASCs at day 7 compared to day 2.

### Memory B-cell responses.

**FIG 4:**
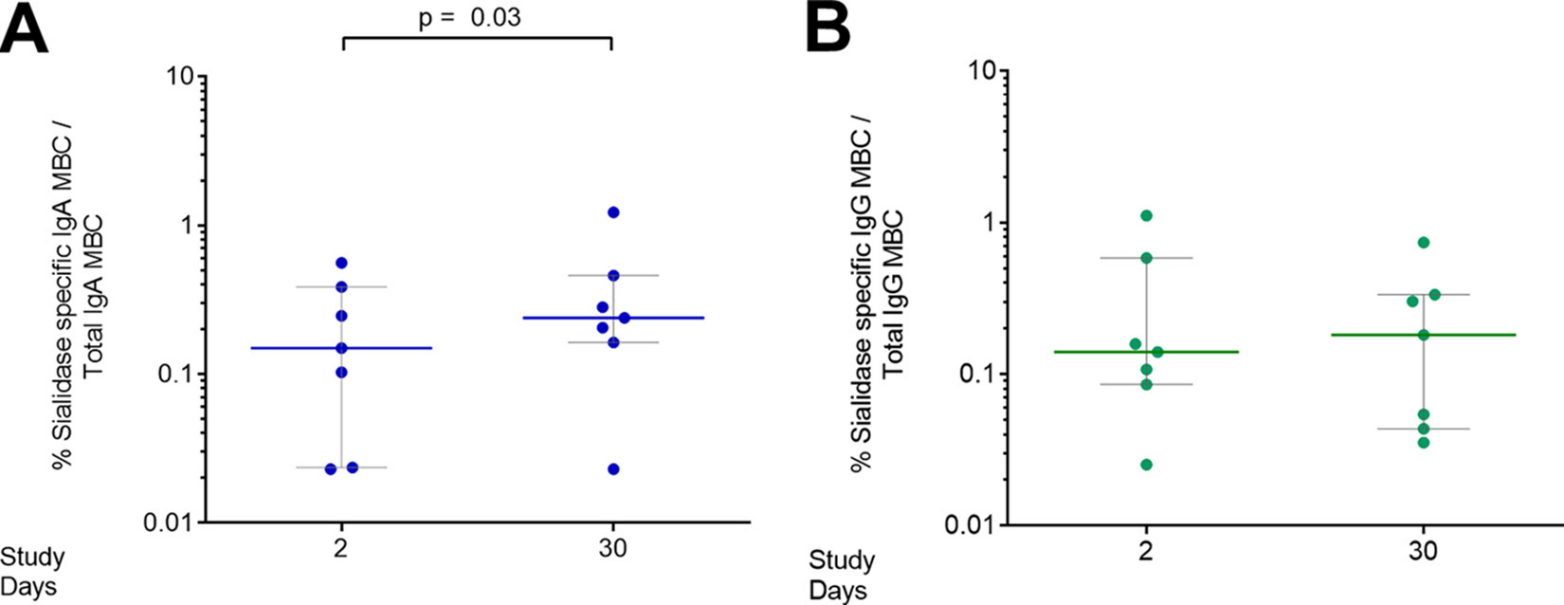
Sialidase-specific memory B-cell (MBC) responses in adult patients with cholera. Shown are the proportions of anti-sialidase IgA (A) and IgG (B) MBCs as a fraction of total MBCs of the same antibody isotype in adults on days 2 and 30 following cholera. Medians with interquartile ranges are shown. Differences within groups were assessed using the Wilcoxon matched-pairs signed-rank test. *P ≤ *0.05 was considered significant.

We measured memory B-cell responses in adult patients (*n* = 7) on days 2 and 30 following cholera (Fig. 4); children were excluded due to the lower volume of blood available for isolation of PBMCs. We found a significant sialidase-specific IgA memory B-cell response at late convalescence (day 30) compared to acute (day 2) stages (*P* = 0.03), but we did not observe a significant increase in IgG memory B-cell response at day 30.

### Antisialidase responses in household contacts.

**FIG 5:**
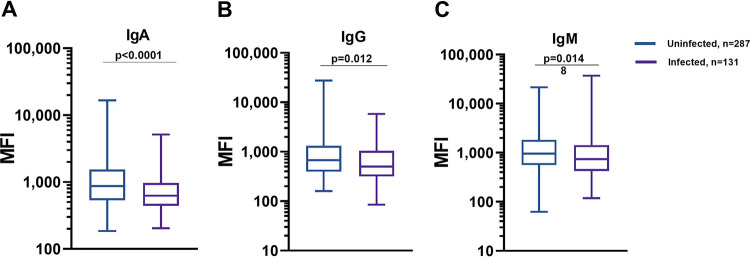
Antisialidase-specific plasma response on exposure in subsequently uninfected and infected household contacts. Shown are box blot diagrams of mean fluorescence intensities (MFI) of plasma IgA (A), IgG (B), and IgM (C) responses of uninfected and infected household contacts. Differences between groups were assessed using the Mann-Whitney U test. *P ≤ *0.05 was considered significant.

We measured plasma anti-sialidase IgA, IgG, and IgM responses in household contacts on the day of enrollment by Luminex. Contacts that remained subsequently uninfected had higher baseline IgA, IgG, and IgM titers than contacts that became infected after exposure to an index case (*P* < 0.0001, *P* = 0.012, and *P* = 0.0148, respectively) (Fig. 5).

## DISCUSSION

The vibriocidal antibody response, measured as the reciprocal titer in serum of complement-dependent killing of V. cholerae, is the best-characterized immune correlate of protection for cholera, where the major antigen is lipopolysaccharide (LPS) (21). However, vibriocidal antibodies in serum disappear much more rapidly than the protection following infection (22), and there is no vibriocidal antibody threshold that correlates with protection (23). Although both natural infection and oral cholera vaccines (inactivated and live attenuated) induce vibriocidal antibody responses, vaccines confer shorter-lived protection than natural infection, especially in young children (22, 24). A better understanding of the longer-term immune correlates of protection against cholera is needed.

Elevated levels of plasma IgA targeting CTB, TcpA, and LPS/OSP are correlated with protection against cholera in settings where cholera is endemic (25); however, plasma Ig levels to these antigens wane more rapidly than protective immunity (26). Anamnestic responses of memory B cells have been hypothesized to mediate long-term protection against cholera. Memory B cells to specific antigens can be quickly stimulated upon reexposure to the pathogen to produce antibodies, and cholera patients have been shown to develop memory B-cell responses to both V. cholerae O antigen, the primary target of vibriocidal antibodies (27), and cholera toxin (CT) following infection (26). While measurable memory B-cell responses to the O antigen of LPS (a T-cell-independent antigen) wane prior to 1 year, those to CT (a T-cell-dependent antigen) and TcpA have been shown to remain elevated through 1 year of follow-up, suggesting T-cell help may be necessary for long-lasting memory B-cell responses (26). Among household contacts of index patients hospitalized with cholera in Bangladesh, V. cholerae O-antigen-specific memory B-cell responses on exposure, but not the memory B-cell response to CT, were associated with protection (28, 29). However, due to a limited understanding of the complete V. cholerae antigen repertoire, it is not clear whether other T-cell-dependent antigens may contribute to durable protection to cholera.

Our current analysis demonstrates that cholera patients develop antibody responses against sialidase following infection, confirming the results of our prior immunoscreens. There was an age-related immune response to sialidase, with all age groups developing an IgM plasma response, while IgG and IgA plasma responses were more robust in older children and adults. In addition, adults developed significant sialidase-specific IgA ASC responses during acute infection consistent with a mucosal immune response; however, responses in children were less robust. This may reflect repeated exposure over time to V. cholerae in this area of high cholera endemicity, even in the absence of symptomatic infection (30), leading to more significant antisialidase antibody levels in adults. Such an age-dependent increase in V. cholerae immune responsiveness, may in part explain the decrease in susceptibility to cholera with age in areas of endemicity (25).

In contrast to sialidase, CTB is an immunodominant antigen. The magnitude of plasma antibody responses and responder frequencies are higher to CTB compared to those to sialidase, and responses to CTB, unlike to sialidase, are comparable across the age spectrum. In addition, all age groups develop significant IgA and IgG ASC and memory B-cell responses to CTB (31). However, memory B-cell response to CTB responses in cholera patients has not been associated with protection (25, 29). In our analysis, we demonstrate that adults with cholera develop IgA memory B cells specific to sialidase. We also show that plasma antibody levels from all three antibody isotypes are higher in magnitude in household contacts protected from infection, thus providing further evidence to support a potential role for antisialidase antibodies in protection from cholera. Future studies are required to determine the duration of antisialidase plasma antibody responses and memory B-cell responses and whether these are independent predictors of protection beyond age, vibriocidal, and anti-OSP responses. This information could also provide value to seroepidemiological studies beyond what is provided currently by vibriocidal and anti-CTB responses (32).

Our study has several limitations. We evaluated immune responses against sialidase in a limited number of patients, and we only assessed antisialidase responses to 1 month following infection. In addition, memory B-cell responses against sialidase were not evaluated in children infected with cholera. Furthermore, all the patients were from a single country of high endemicity, Bangladesh. It will be important to evaluate antisialidase responses in larger cohorts of patients from different areas where cholera is endemic to confirm our findings and to assess the specificity of this response. By sequence analysis, the V. cholerae sialidase is antigenically distinct from the neuraminidases from other bacterial and viral organisms. However, future investigations are required to confirm and ensure specificity.

In order to further evaluate the potential role of sialidase in longer-term protection from cholera, we should also examine whether antisialidase immune responses are elicited by (i) oral whole-cell killed vaccine and/or (ii) live attenuated vaccine T-cell responses to this protein antigen, which might give insight into the possible development of both T-cell memory and B-cell memory. In addition, although we measured circulating ASCs to sialidase following cholera, more direct measures of antisialidase mucosal responses in gut biopsy specimens and examination for either memory B cells or long-lived plasma cells specific for sialidase in such biopsy specimens would be helpful.

Despite these limitations, this study demonstrates that patients following cholera develop serum/plasma, mucosal, and memory B-cell responses to V. cholerae sialidase and that the pattern of immune responses is consistent with higher responses in adults than younger patients, similar to the age-dependent decrease in susceptibility to cholera seen in areas of endemicity. This suggests that further studies of immune responses to sialidase, including T-cell responses following infection and cholera vaccination, may be warranted.

## MATERIALS AND METHODS

### Study design and specimen collection.

Diarrheal patients hospitalized with cholera at the icddr,b hospital in Dhaka were enrolled following informed consent. Infected patients with V. cholerae O1 as the sole pathogen in stool (19, 20) (index case) were enrolled in this study. Blood specimens were collected from patients at day 2 after hospitalization. Additional blood specimens were collected on days 7 (6 to 9 days later) and 30 (28 to 32 days later).

Household contacts were enrolled and evaluated as previously described (25, 28, 29, 33). Briefly, contacts were enrolled within 24 h after presentation of the cholera index case if they shared the same cooking pot with the index case for 3 or more days prior to the cholera episode. Contacts were monitored for 9 days to evaluate for diarrheal symptoms, presence of V. cholerae O1 in stool, and immune responses to V. cholerae on days 2 and 7. Household contacts were defined as “infected” if any stool culture during the 9 days of follow-up was positive for V. cholerae. Contacts were defined as “uninfected” if they had no diarrhea, all stool cultures were negative for V. cholerae, and they did not have a 4-fold rise in their vibriocidal titer during the follow-up period. We excluded contacts who did not fall into one of these two categories, did not complete follow-up, had any diarrhea the week prior to enrollment, or had V. cholerae with a different serogroup or serotype than the corresponding index case.

### Ethics statement.

This study was approved by the institutional review boards of icddr,b and Massachusetts General Hospital. Informed written consent was obtained from all study participants.

### Isolation of plasma and PBMCs.

Heparinized venous blood was diluted in phosphate-buffered saline (PBS; pH 7.2 to 7.4); peripheral blood mononuclear cells (PBMCs) and plasma were separated by density gradient centrifugation using Ficoll-Isopaque (Pharmacia, Piscataway, NJ). Isolated plasma specimens were frozen at −20°C prior to use in immunological assays. PBMCs were suspended in RPMI complete medium (Gibco, Carlsbad, CA) containing 10% heat-inactivated fetal bovine serum (FBS; HyClone, Logan, UT) at a concentration of 1 × 10^7^ cells/ml and used immediately for assays.

### Vibriocidal antibody assay.

Vibriocidal antibody titers were measured as described previously using V. cholerae O1 Ogawa (strain X25049) and Inaba (strain T19479) as the target organisms (34). The vibriocidal titer has been defined as the reciprocal of the maximum dilution of plasma/serum resulting in a ≥50% drop of the absorbance compared to that of control wells without plasma/serum. Any volunteer who had a ≥4-fold increase of vibriocidal titer from baseline (day 2) level was considered a responder.

### Purification of V. cholerae sialidase.

Sialidase was purified as previously described (11). Briefly, sialidase was overexpressed in Escherichia coli as a recombinant polyhistidine protein, purified by affinity chromatography using HisPur Cobalt spin columns (Thermo Scientific Pierce) under denaturing conditions, and refolded by dialysis into 25 mM Tris-HCl (pH 8.0) with 0.15 M sodium chloride. The purity of the refolded sialidase was assessed by SDS-PAGE and quantified by Pierce Coomassie Plus (Bradford) assay reagent (Thermo Fisher Scientific, Waltham, MA).

### Sialidase and CTB-specific IgA, IgG, and IgM antibodies in plasma.

Plasma sialidase and CTB-specific IgA, IgG, and IgM responses were assessed using modification of enzyme-linked immunosorbent assay (ELISA) protocols (35, 36). Briefly, 96-well polystyrene plates (Nunc F, Denmark) were coated with sialidase (2.5 μg/ml) and then blocked with 1% bovine serum albumin (BSA; Sigma, St. Louis, MO) in phosphate-buffered saline (PBS). We then added a 1:25 dilution of plasma (100 μl/well), followed by horseradish peroxidase-conjugated anti-human immunoglobulin antibodies of the relevant isotype (1:1,000 dilution; Jackson ImmunoResearch, West Grove, PA). We detected bound antibody with *ortho*-phenylenediamine (OPD; Sigma, St. Louis, MO) in 0.1 M sodium citrate buffer (pH 4.5) and 0.012% hydrogen peroxide. Optical density was measured at 450 nm for 5 min, and the rate of change in optical density was measured as milli-absorbance units per minute. The absorbance value of each sample was then normalized and expressed as ELISA units by calculating the ratio of the sample to a standard of pooled plasma. A positive response was defined as 1.5-fold increase in ELISA units from day 2 to convalescence (either day 7 or day 30).

### Antibody-secreting cell assay.

Antibody-secreting cells were measured in peripheral blood according to our previously described enzyme-linked immunosorbent spot (ELISPOT) procedure (26, 37). Briefly, nitrocellulose (bottom) plates (MSHAN-4550; Millipore, Bedford, MA) were coated with 100 μl of sialidase (25 μg/ml) in sodium bicarbonate buffer (pH 9.6), affinity-purified goat anti-human immunoglobulin (5 μg/ml; Jackson Immuno Research, West Grove, PA) to detect the total number of circulating ASCs, or keyhole limpet hemocyanin (KLH; 2.5 μg/ml [Pierce Biotechnology, Rockford, IL]) in PBS as a negative control. After blocking with RPMI solution, isolated PBMCs were incubated on the plates for 3 h, and secreted antibodies were detected with peroxidase-conjugated mouse anti-human IgA (1:500 dilution; Southern Biotech, Birmingham, AL) and alkaline phosphatase-conjugated IgG (1:500 dilution; Southern Biotech, Birmingham, AL). ASCs were detected with 3-amino-9-ethylcarbazole (AEC) (AEC premix solution; Sigma-Aldrich) and 5-bromo-4-chloro-3-indolylphosphate–nitroblue tetrazolium (BCIP/NBT; Sigma-Aldrich). The antigen-specific IgA and IgG isotypes of ASCs were expressed as the frequencies of the total circulating ASCs of the same isotype at the same time point.

### Memory B-cell culture and ELISPOT assay.

We used a previously published procedure to detect and quantify antigen-specific memory B cells in circulation (26, 28). In brief, isolated PBMCs were plated in 24-well cell culture plates (BD Biosciences, San Jose, CA) at a concentration of 5 × 10^5^ PBMCs/well. Cells were stimulated for proliferation and differentiation of memory B cells into ASCs for 5 to 6 days in an antigen-independent manner using a mixture of B-cell mitogens, except those being used as negative controls, to which only medium was added. After stimulation, harvested cells were incubated for 5 h on ELISPOT plates coated with sialidase, affinity-purified goat anti-human immunoglobulin, or KLH as described above. Alkaline phosphatase-conjugated goat anti-human IgG (Southern Biotech, Birmingham, AL) and horseradish peroxidase-conjugated mouse anti-human IgA (Southern Biotech, Birmingham, AL) were added at a dilution of 1:500. Following an overnight incubation at 4°C, plates were developed with 5-bromo-4-chloro-3-indolylphosphate (BCIP)–nitroblue tetrazolium (BCIP/NBT) for IgG and with 3-amino-9-ethyl carbazole (AEC) for IgA. Sialidase-specific memory B cells were expressed as the percentage of antigen-specific memory B cells out of the total isotype-specific memory B cells present at the same time point. We excluded data from our analysis if (i) the total Ig sample did not have appropriate stimulation, (ii) patient samples had three or more antigen-specific ASC spots prior to stimulation, or (iii) patient samples had three or more ASC spots to the negative-control antigen KLH after stimulation (26, 28). Appropriate stimulation of PBMCs in our assay was defined as a >3-fold increase in the number of total Ig memory cells following stimulation compared to unstimulated cells. The limits of detection for sialidase-specific IgA and IgG MBCs were 0.004 and 0.001%, respectively, per 5 × 10^5^ PBMCs after 6 days of stimulation (26).

### Luminex assay.

Antisialidase responses were measured in protected and unprotected cohorts of household contacts by Luminex. Sialidase was conjugated to phycoerythrin (PE)-labeled MagPlex-C microspheres (Luminex Corp., Austin, TX) at a concentration of 5 μg of antigen per 1 × 10^6^ microspheres per the manufacturer’s instructions. Plasma samples were heat inactivated by incubation at 56°C for 30 min and diluted with assay buffer (0.1% BSA in 1× PBS) to a final dilution of 1:100. Five microliters of diluted plasma and 45 μl of sialidase-conjugated beads were added to a black 384-well polystyrene plate (Greiner Bio-One, Monroe, NC) at a final concentration of 15 beads per μl (total of 675 beads per well). Plates were incubated overnight at 4°C with shaking (800 rpm) and then sonicated and washed with 0.1% BSA in 1× PBS–0.05% Tween 20 before addition of PE-conjugated anti-human IgG, IgM, and IgA (40 μl per well; Southern Biotech) for a final dilution of 1:154. After incubation at 1 h with shaking, plates were sonicated and washed. The beads were resuspended in 40 μl of sheath fluid (Fisher Scientific, Waltham, MA) and analyzed using either Bioplex or Flexmap machines (Bio-Rad). Samples were tested in duplicate, and data are presented as the average of the mean fluorescence intensity of the duplicates.

### Statistical analyses.

We assessed differences in the magnitudes of responses using the Wilcoxon signed-rank test or the Mann-Whitney U test, as appropriate. Normality of Luminex data was tested using the Shapiro-Wilk test. chi-square tests were used to assess relative risk ratios with confidence intervals. All reported *P* values were two-tailed, with a cutoff of *P ≤ *0.05 considered a threshold for statistical significance. We performed analysis using GraphPad Prism 6.0 (GraphPad Software, Inc., La Jolla, CA).

## References

[1] Harris JB, LaRocque RC, Qadri F, Ryan ET, Calderwood SB. 2012. Cholera. Lancet 379:2466–2476. doi:10.1016/S0140-6736(12)60436-X. 22748592 PMC3761070

[2] Qadri F, Islam T, Clemens JD. 2017. Cholera in Yemen—an old foe rearing its ugly head. N Engl J Med 377:2005–2007. doi:10.1056/NEJMp1712099. 29091747

[3] Deen J, Mengel MA, Clemens JD. 2020. Epidemiology of cholera. Vaccine 38(Suppl 1):A31–A40. doi:10.1016/j.vaccine.2019.07.078. 31395455

[4] Ali M, Nelson AR, Lopez AL, Sack DA. 2015. Updated global burden of cholera in endemic countries. PLoS Negl Trop Dis 9:e0003832. doi:10.1371/journal.pntd.0003832. 26043000 PMC4455997

[5] Bhattacharya SK, Sur D, Ali M, Kanungo S, You YA, Manna B, Sah B, Niyogi SK, Park JK, Sarkar B, Puri MK, Kim DR, Deen JL, Holmgren J, Carbis R, Dhingra MS, Donner A, Nair GB, Lopez AL, Wierzba TF, Clemens JD. 2013. 5 year efficacy of a bivalent killed whole-cell oral cholera vaccine in Kolkata, India: a cluster-randomised, double-blind, placebo-controlled trial. Lancet Infect Dis 13:1050–1056. doi:10.1016/S1473-3099(13)70273-1. 24140390

[6] Clemens JD, Sack DA, Harris JR, Van Loon F, Chakraborty J, Ahmed F, Rao MR, Khan MR, Yunus M, Huda N. 1990. Field trial of oral cholera vaccines in Bangladesh: results from three-year follow-up. Lancet 335:270–273. doi:10.1016/0140-6736(90)90080-o. 1967730

[7] van Loon FP, Clemens JD, Chakraborty J, Rao MR, Kay BA, Sack DA, Yunus M, Ali M, Svennerholm AM, Holmgren J. 1996. Field trial of inactivated oral cholera vaccines in Bangladesh: results from 5 years of follow-up. Vaccine 14:162–166. doi:10.1016/0264-410X(95)00122-H. 8852414

[8] Glass RI, Becker S, Huq MI, Stoll BJ, Khan MU, Merson MH, Lee JV, Black RE. 1982. Endemic cholera in rural Bangladesh, 1966–1980. Am J Epidemiol 116:959–970. doi:10.1093/oxfordjournals.aje.a113498. 7148820

[9] Levine MM, Black RE, Clements ML, Cisneros L, Nalin DR, Young CR. 1981. Duration of infection-derived immunity to cholera. J Infect Dis 143:818–820. doi:10.1093/infdis/143.6.818. 7252264

[10] Kauffman RC, Bhuiyan TR, Nakajima R, Mayo-Smith LM, Rashu R, Hoq MR, Chowdhury F, Khan AI, Rahman A, Bhaumik SK, Harris L, O'Neal JT, Trost JF, Alam NH, Jasinskas A, Dotsey E, Kelly M, Charles RC, Xu P, Kovac P, Calderwood SB, Ryan ET, Felgner PL, Qadri F, Wrammert J, Harris JB. 2016. Single-cell analysis of the plasmablast response to Vibrio cholerae demonstrates expansion of cross-reactive memory B cells. mBio 7:e02021-16. doi:10.1128/mBio.02021-16. 27999163 PMC5181778

[11] Charles RC, Nakajima R, Liang L, Jasinskas A, Berger A, Leung DT, Kelly M, Xu P, Kovac P, Giffen SR, Harbison JD, Chowdhury F, Khan AI, Calderwood SB, Bhuiyan TR, Harris JB, Felgner PL, Qadri F, Ryan ET. 2017. Plasma and mucosal immunoglobulin M, immunoglobulin A, and immunoglobulin G responses to the Vibrio cholerae O1 protein immunome in adults with cholera in Bangladesh. J Infect Dis 216:125–134. doi:10.1093/infdis/jix253. 28535267 PMC5853614

[12] Corfield AP, Higa H, Paulson JC, Schauer R. 1983. The specificity of viral and bacterial sialidases for alpha(2-3)- and alpha(2-6)-linked sialic acids in glycoproteins. Biochim Biophys Acta 744:121–126. doi:10.1016/0167-4838(83)90080-8. 6301560

[13] Crennell S, Garman E, Laver G, Vimr E, Taylor G. 1994. Crystal structure of Vibrio cholerae neuraminidase reveals dual lectin-like domains in addition to the catalytic domain. Structure 2:535–544. doi:10.1016/s0969-2126(00)00053-8. 7922030

[14] Moustafa I, Connaris H, Taylor M, Zaitsev V, Wilson JC, Kiefel MJ, von Itzstein M, Taylor G. 2004. Sialic acid recognition by Vibrio cholerae neuraminidase. J Biol Chem 279:40819–40826. doi:10.1074/jbc.M404965200. 15226294

[15] Heidelberg JF, Eisen JA, Nelson WC, Clayton RA, Gwinn ML, Dodson RJ, Haft DH, Hickey EK, Peterson JD, Umayam L, Gill SR, Nelson KE, Read TD, Tettelin H, Richardson D, Ermolaeva MD, Vamathevan J, Bass S, Qin H, Dragoi I, Sellers P, McDonald L, Utterback T, Fleishmann RD, Nierman WC, White O, Salzberg SL, Smith HO, Colwell RR, Mekalanos JJ, Venter JC, Fraser CM. 2000. DNA sequence of both chromosomes of the cholera pathogen Vibrio cholerae. Nature 406:477–483. doi:10.1038/35020000. 10952301 PMC8288016

[16] Jermyn WS, Boyd EF. 2005. Molecular evolution of Vibrio pathogenicity island-2 (VPI-2): mosaic structure among Vibrio cholerae and Vibrio mimicus natural isolates. Microbiology (Reading) 151:311–322. doi:10.1099/mic.0.27621-0. 15632448

[17] Galen JE, Ketley JM, Fasano A, Richardson SH, Wasserman SS, Kaper JB. 1992. Role of Vibrio cholerae neuraminidase in the function of cholera toxin. Infect Immun 60:406–415. doi:10.1128/IAI.60.2.406-415.1992. 1730470 PMC257643

[18] Almagro-Moreno S, Boyd EF. 2009. Sialic acid catabolism confers a competitive advantage to pathogenic vibrio cholerae in the mouse intestine. Infect Immun 77:3807–3816. doi:10.1128/IAI.00279-09. 19564383 PMC2738016

[19] Rahman M, Sack DA, Mahmood S, Hossain A. 1987. Rapid diagnosis of cholera by coagglutination test using 4-h fecal enrichment cultures. J Clin Microbiol 25:2204–2206. doi:10.1128/JCM.25.11.2204-2206.1987. 3693549 PMC269441

[20] Schwartz BS, Harris JB, Khan AI, Larocque RC, Sack DA, Malek MA, Faruque AS, Qadri F, Calderwood SB, Luby SP, Ryan ET. 2006. Diarrheal epidemics in Dhaka, Bangladesh, during three consecutive floods: 1988, 1998, and 2004. Am J Trop Med Hyg 74:1067–1073. doi:10.4269/ajtmh.2006.74.1067. 16760521 PMC1626162

[21] Haney DJ, Lock MD, Simon JK, Harris J, Gurwith M. 2017. Antibody-based correlates of protection against cholera analysis of a challenge study in a cholera-naive population. Clin Vaccine Immunol 24:e00098-17. doi:10.1128/CVI.00098-17. PMC5583470 28566334

[22] Alam MM, Riyadh MA, Fatema K, Rahman MA, Akhtar N, Ahmed T, Chowdhury MI, Chowdhury F, Calderwood SB, Harris JB, Ryan ET, Qadri F. 2011. Antigen-specific memory B-cell responses in Bangladeshi adults after one- or two-dose oral killed cholera vaccination and comparison with responses in patients with naturally acquired cholera. Clin Vaccine Immunol 18:844–850. doi:10.1128/CVI.00562-10. 21346055 PMC3122537

[23] Saha D, LaRocque RC, Khan AI, Harris JB, Begum YA, Akramuzzaman SM, Faruque AS, Ryan ET, Qadri F, Calderwood SB. 2004. Incomplete correlation of serum vibriocidal antibody titer with protection from Vibrio cholerae infection in urban Bangladesh. J Infect Dis 189:2318–2322. doi:10.1086/421275. 15181581

[24] Rahman A, Rashu R, Bhuiyan TR, Chowdhury F, Khan AI, Islam K, LaRocque RC, Ryan ET, Wrammert J, Calderwood SB, Qadri F, Harris JB. 2013. Antibody-secreting cell responses after Vibrio cholerae O1 infection and oral cholera vaccination in adults in Bangladesh. Clin Vaccine Immunol 20:1592–1598. doi:10.1128/CVI.00347-13. 23945156 PMC3807192

[25] Harris JB, LaRocque RC, Chowdhury F, Khan AI, Logvinenko T, Faruque AS, Ryan ET, Qadri F, Calderwood SB. 2008. Susceptibility to Vibrio cholerae infection in a cohort of household contacts of patients with cholera in Bangladesh. PLoS Negl Trop Dis 2:e221. doi:10.1371/journal.pntd.0000221. 18398491 PMC2271133

[26] Harris AM, Bhuiyan MS, Chowdhury F, Khan AI, Hossain A, Kendall EA, Rahman A, LaRocque RC, Wrammert J, Ryan ET, Qadri F, Calderwood SB, Harris JB. 2009. Antigen-specific memory B-cell responses to Vibrio cholerae O1 infection in Bangladesh. Infect Immun 77:3850–3856. doi:10.1128/IAI.00369-09. 19528207 PMC2738048

[27] Johnson RA, Uddin T, Aktar A, Mohasin M, Alam MM, Chowdhury F, Harris JB, LaRocque RC, Bufano MK, Yu Y, Wu-Freeman Y, Leung DT, Sarracino D, Krastins B, Charles RC, Xu P, Kovac P, Calderwood SB, Qadri F, Ryan ET. 2012. Comparison of immune responses to the O-specific polysaccharide and lipopolysaccharide of Vibrio cholerae O1 in Bangladeshi adult patients with cholera. Clin Vaccine Immunol 19:1712–1721. doi:10.1128/CVI.00321-12. 22993410 PMC3491541

[28] Aktar A, Rahman MA, Afrin S, Akter A, Uddin T, Yasmin T, Sami MIN, Dash P, Jahan SR, Chowdhury F, Khan AI, LaRocque RC, Charles RC, Bhuiyan TR, Mandlik A, Kelly M, Kovac P, Xu P, Calderwood SB, Harris JB, Qadri F, Ryan ET. 2018. Plasma and memory B cell responses targeting O-specific polysaccharide (OSP) are associated with protection against Vibrio cholerae O1 infection among household contacts of cholera patients in Bangladesh. PLoS Negl Trop Dis 12:e0006399. doi:10.1371/journal.pntd.0006399. 29684006 PMC5912711

[29] Patel SM, Rahman MA, Mohasin M, Riyadh MA, Leung DT, Alam MM, Chowdhury F, Khan AI, Weil AA, Aktar A, Nazim M, LaRocque RC, Ryan ET, Calderwood SB, Qadri F, Harris JB. 2012. Memory B cell responses to Vibrio cholerae O1 lipopolysaccharide are associated with protection against infection from household contacts of patients with cholera in Bangladesh. Clin Vaccine Immunol 19:842–848. doi:10.1128/CVI.00037-12. 22518009 PMC3370438

[30] Weil AA, Chowdhury F, Khan AI, Leung DT, Uddin T, Begum YA, Saha NC, Charles RC, Larocque RC, Harris JB, Ryan ET, Qadri F, Calderwood SB. 2012. Frequency of reexposure to Vibrio cholerae O1 evaluated by subsequent vibriocidal titer rise after an episode of severe cholera in a highly endemic area in Bangladesh. Am J Trop Med Hyg 87:921–926. doi:10.4269/ajtmh.2012.12-0323. 22964723 PMC3516269

[31] Leung DT, Rahman MA, Mohasin M, Riyadh MA, Patel SM, Alam MM, Chowdhury F, Khan AI, Kalivoda EJ, Aktar A, Bhuiyan MS, LaRocque RC, Harris JB, Calderwood SB, Qadri F, Ryan ET. 2011. Comparison of memory B cell, antibody-secreting cell, and plasma antibody responses in young children, older children, and adults with infection caused by Vibrio cholerae O1 El Tor Ogawa in Bangladesh. Clin Vaccine Immunol 18:1317–1325. doi:10.1128/CVI.05124-11. 21697337 PMC3147357

[32] Azman AS, Lessler J, Luquero FJ, Bhuiyan TR, Khan AI, Chowdhury F, Kabir A, Gurwith M, Weil AA, Harris JB, Calderwood SB, Ryan ET, Qadri F, Leung DT. 2019. Estimating cholera incidence with cross-sectional serology. Sci Transl Med 11:eaau6242. doi:10.1126/scitranslmed.aau6242. 30787170 PMC6430585

[33] Weil AA, Begum Y, Chowdhury F, Khan AI, Leung DT, LaRocque RC, Charles RC, Ryan ET, Calderwood SB, Qadri F, Harris JB. 2014. Bacterial shedding in household contacts of cholera patients in Dhaka, Bangladesh. Am J Trop Med Hyg 91:738–742. doi:10.4269/ajtmh.14-0095. 25114012 PMC4183396

[34] Qadri F, Mohi G, Hossain J, Azim T, Khan AM, Salam MA, Sack RB, Albert MJ, Svennerholm AM. 1995. Comparison of the vibriocidal antibody response in cholera due to Vibrio cholerae O139 Bengal with the response in cholera due to Vibrio cholerae O1. Clin Diagn Lab Immunol 2:685–688. doi:10.1128/CDLI.2.6.685-688.1995. 8574829 PMC170220

[35] Qadri F, Ahmed F, Karim MM, Wenneras C, Begum YA, Abdus Salam M, Albert MJ, McGhee JR. 1999. Lipopolysaccharide- and cholera toxin-specific subclass distribution of B-cell responses in cholera. Clin Diagn Lab Immunol 6:812–818. doi:10.1128/CDLI.6.6.812-818.1999. 10548569 PMC95781

[36] Qadri F, Wenneras C, Albert MJ, Hossain J, Mannoor K, Begum YA, Mohi G, Salam MA, Sack RB, Svennerholm AM. 1997. Comparison of immune responses in patients infected with Vibrio cholerae O139 and O1. Infect Immun 65:3571–3576. doi:10.1128/IAI.65.9.3571-3576.1997. 9284121 PMC175508

[37] Qadri F, Ryan ET, Faruque AS, Ahmed F, Khan AI, Islam MM, Akramuzzaman SM, Sack DA, Calderwood SB. 2003. Antigen-specific immunoglobulin A antibodies secreted from circulating B cells are an effective marker for recent local immune responses in patients with cholera: comparison to antibody-secreting cell responses and other immunological markers. Infect Immun 71:4808–4814. doi:10.1128/iai.71.8.4808-4814.2003. 12874365 PMC165990

