# Supplementary figures and images for: Vibrio cholerae Sialidase-Specific Immune Responses Are Associated with Protection against Cholera
Source: mSphere. 2021 Apr 28;6(2):e01232-20. doi: 10.1128/mSphere.01232-20 (PMC8092141; doi:10.1128/mSphere.01232-20)

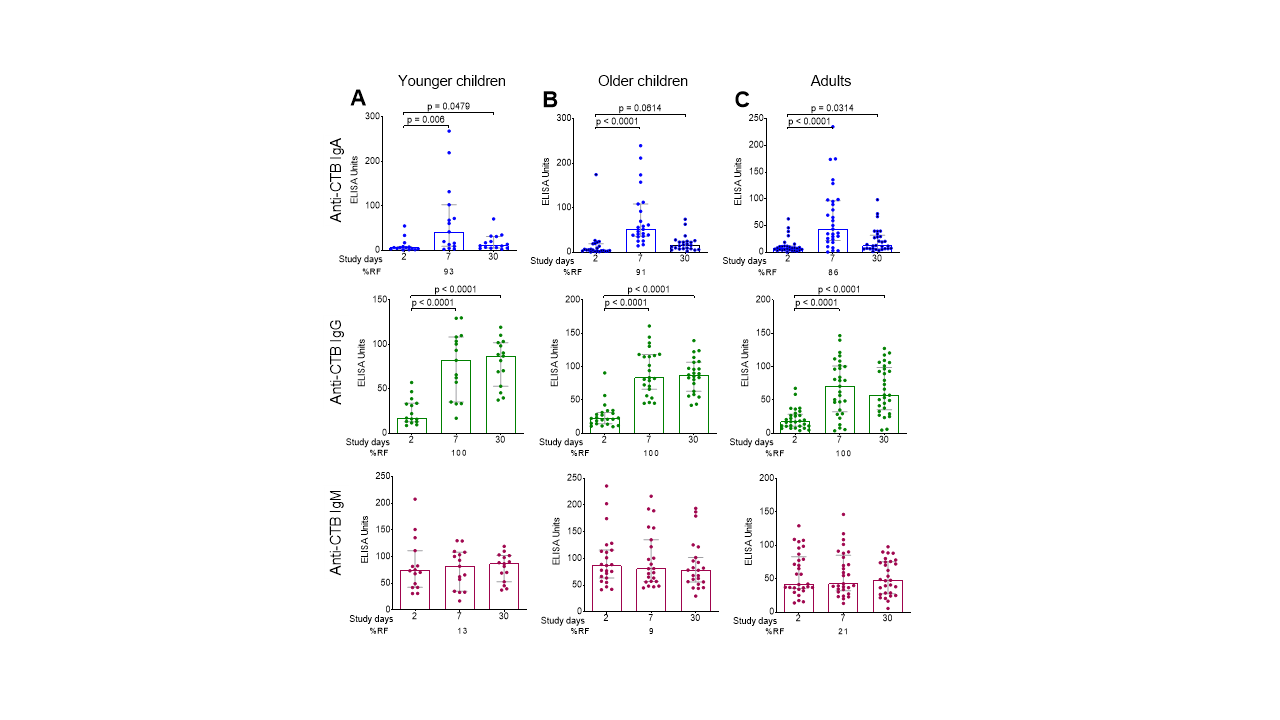

Supplement: FIG S1 [file mSphere.01232-20-sf001.tif]
